# Challenges and strategies for conducting research in primary health care practice: an integrative review

**DOI:** 10.1186/s12913-023-10382-1

**Published:** 2023-12-08

**Authors:** Daiana Bonfim, Lorrayne Belotti, Leticia Yamawaka de Almeida, Ilana Eshriqui, Sofia Rafaela Maito Velasco, Camila Nascimento Monteiro, Adelson Guaraci Jantsch

**Affiliations:** 1 Hospital Israelita Albert Einstein - Albert Einstein Center for Studies, Research, and Practices in Primary Health Care and Networks, Sao Paulo, Brazil; 2 Executive Secretariat of Organization Open University of the Unified Health System (UNASUS), Brasilia, Brazil

**Keywords:** Primary health care, Research, Review, Health personnel

## Abstract

**Background:**

Providing accessible and high-quality patient-centered healthcare remains a challenge in many countries, despite global efforts to strengthen primary health care (PHC). Research and knowledge management are integral to enhancing PHC, facilitating the implementation of successful strategies, and promoting the use of evidence-based practices. Practice-based research in primary care (PC-PBR) has emerged as a valuable approach, with its external validity to diverse PHC settings, making it an effective means of translating research findings into professional practice.

**Objective:**

To identify challenges and strategies for conducting practice-based research in primary health care services.

**Method:**

An integrative literature review was conducted by searching the PubMed, Embase, Scopus, Web of Science, and Lilacs databases. The research question, guided by the PICo framework, directed the execution of study selection and data extraction. Data analysis followed the RAdAR method's three phases: pre-analysis, data analysis, and interpretation of results.

**Results:**

Out of 440 initially identified articles, 26 met the inclusion criteria. Most studies were conducted in high-income countries, primarily the United States. The challenges and strategies for PC-PBR were categorized into six themes: research planning, infrastructure, engagement of healthcare professionals, knowledge translation, the relationship between universities and health services, and international collaboration. Notable challenges included research planning complexities, lack of infrastructure, difficulties in engaging healthcare professionals, and barriers to knowledge translation. Strategies underscore the importance of adapting research agendas to local contexts, providing research training, fostering stakeholder engagement, and establishing practice-based research networks.

**Conclusion:**

The challenges encountered in PC-PBR are consistent across various contexts, highlighting the need for systematic, long-term actions involving health managers, decision-makers, academics, diverse healthcare professionals, and patients. This approach is essential to transform primary care, especially in low- and middle-income countries, into an innovative, comprehensive, patient-centered, and accessible healthcare system. By addressing these challenges and implementing the strategies, PC-PBR can play a pivotal role in bridging the gap between research and practice, ultimately improving patient care and population health.

## Introduction

Despite global efforts toward strengthening primary health care (PHC) in the last 40 years, providing accessible and good quality patient-centered health care is still a challenge to most countries. Recently, the report *Operational Framework for Primary Health Care* (2020) released by the World Health Organization reinforced the principles of the Astana Declaration highlighting 14 levers that must be simultaneously pulled to promote PHC across the world [1].

One of those 14 “operational levers” describes the importance of conducting research that is meaningful for PHC: “*Research and knowledge management, including dissemination of lessons learned, as well as the use of knowledge to accelerate the scale-up of successful strategies to strengthen PHC*” [1]*.* Although conducting research that meets these premises is not simple, primary care practice-based research (PC-PBR) has become an important vehicle for the development of science in the real world, because of its external validity to other PHC settings and contexts, making knowledge translation easier to put evidence into professional practice [2].

PC-PBR occurs in the context of patient health care in the community, according to Dolor et al. (2015), resulting in the research questions being primarily generated by the health services to respond to the needs of their territory [3]. PHC is responsible for serving as the first point of contact for patients, through which all health issues should be addressed. It serves as an ideal setting for conducting practice-based research, encompassing the implementation of innovations and studies aimed at enhancing the quality of care for various health conditions. These conditions span across diverse areas, including mental health [4] and chronic kidney disease [5]. Furthermore, it is also pertinent in the context of public health emergencies, such as the COVID-19 pandemic [6].

One solution to foster this type of research is creating practice-based research networks (PBRNs). Their aim is to bring healthcare professionals, researchers, health managers, and academic institutions together, facilitating partnerships, and providing structure and technical support to healthcare professionals to carry out research projects that are developed and conducted in PHC settings to tackle important aspects of PHC [7, 8]. They also help on the job of acquiring funding, capacity building, organizing the necessary logistics to put a research project in place and all sorts of tasks from study design to publication [3, 9]. In this way, PBRNs seek to promote a culture of scientific research in an environment originally dedicated to health care [10] and to answer relevant questions about the local health needs of PHC services. According to Bodenheimer et al. (2005), PBRNs are increasingly seen as institutions that can simultaneously conduct research efficiently and leverage changes in practice [11], serving as laboratories for approaching important challenges to PHC.

However, a preview study [9] developed in Canada described some lessons learned to engage PBRLNs present aspects related to the need for continuity in ethics, regular team meetings, enhancing levels of engagement with stakeholders, the need for structural support and recognizing differences in data sharing across provinces.

Even though the literature on PC-PBR is growing, “How to implement a PBRN and how to scale PC-PBR?” and “How can a healthcare service become a setting for knowledge and innovation production?” are two questions still unanswered. Moreover, scenarios with incipient PHC could benefit from evidence-oriented policies and practice-oriented research. To answer these two questions, available information from places that already run PC-PBR projects needs to be systematized around the challenges, obstacles and solutions found by other researchers. Aiming to help researchers from low- and middle-income countries that are willing to produce research in primary care, we performed an integrative review identifying the challenges and strategies for carrying out PC-PBR.

## Methods

An integrative literature review was performed based on the methodology proposed by Whittemore & Knafl (2005) [12] that includes (a) identification of the problem, (b) literature search, (c) evaluation, (d) analysis and (e) presentation of results. Differently from a systematic review, the broader focus of an integrative review enables the inclusion of studies using different methodologies (qualitative, quantitative and mixed) in the analysis and supplies the methodological rigor necessary for a broader understanding of one specific phenomenon [13, 14].

### Literature search

The research question was developed using the PICo framework (Population, Interest and Context). The elements were organized by P - Primary health care (PHC); I - Challenges and Strategies; Co - Practice-based research (PBR); resulting in the guiding question: “What are the challenges and strategies to carry out PBR in PHC?”. Data were collected in February 2022 by a librarian affiliated with the authors' institution from the databases PubMed, Embase, Scopus, Web of Science, and Lilacs. The database selection was conducted to ensure comprehensive coverage of relevant literature, encompassing multidisciplinary and geographical perspectives related to practice-based research in primary care. The search utilized descriptions and keywords from the Medical Subject Headings (MeSH) and Health Science Descriptors (DeCS), combined with the Boolean operators 'AND' and 'OR' (Table 1).

**Table 1:** Search strategies, according to the database and Boolean operators

**Database**	**Search Strategies**
Scopus	(KEY ("Primary care" OR "community-based care" OR "community-based PHC" ) AND TITLE-ABS-KEY ("family practice research" OR "practice based research" OR "service research") AND KEY ("barriers" OR "challenges" OR "capacity building"))t
Pubmed	(Primary care [Title/Abstract] OR community-based care [Title/Abstract] OR community-based PHC [Title/Abstract]) AND (family practice research [Title/Abstract] OR practice based research [Title/Abstract] OR service research [Title/Abstract]) AND (barriers[Title/Abstract] OR challenges [Title/Abstract] OR capacity building [Title/Abstract])
Embase	('primary care':ti,ab,kw OR 'community-based care':ti,ab,kw OR 'community-based phc':ti,ab,kw) AND ('family practice research':ti,ab,kw OR 'practice based research':ti,ab,kw OR 'service research':ti,ab,kw) AND ('barriers':ti,ab,kw OR 'challenges':ti,ab,kw OR 'capacity building':ti,ab,kw)
Web of science	Primary care OR community-based care OR community-based PHC (Author Keywords) and family practice research OR practice based research OR service research (Topic) and barriers OR challenges OR capacity building (Author Keywords)
Lilacs	(Primary care) AND (research) AND (based) AND (practice)

### Study selection

Articles in English, Spanish and Portuguese were included, regardless of their publication year. Review studies, essays, letters to the editor, studies conducted in non-PHC settings (e.g., emergency services), and those focused on specific health problems were excluded.

Two researchers independently screened the articles by title and abstract (SRMV e AGJ), and the disagreements were resolved through discussion and mediation by a third author (LB). Following this stage, the studies were read in their entirety by the same two authors. During this phase, any remaining disagreements regarding the final inclusion were examined and decided by the authors. In the study selection phase, the software Rayyan was employed as a tool for managing and screening research articles.

### Data extraction

Information was systematically extracted from the selected articles and organized using a custom-designed spreadsheet, enabling the identification of key aspects essential for addressing the research question. These included author names, publication year, study type, study location, research objectives, methodologies employed, study populations, primary internal and external challenges encountered in operationalizing research within primary healthcare, and strategies offered for its effective implementation.

### Data synthesis

The review followed a deductive approach that prioritized the extraction and summarization of studies included as the primary objective of the review and synthesis [15]. This process entails extracting the results from each included paper and categorizing them according to common themes or meanings. These categories are subsequently further organized, allowing for a summary that yields synthesized findings: practical and actionable guidelines suitable for informing policy and formulating strategies [16].

To achieve this, the data analysis followed the steps established by the three distinct phases of the RADaR method: pre-analysis, data analysis, and interpretation of the results [17]. In the pre-analysis stage, each article was read, and its information was extracted and stored in a spreadsheet created to summarize all articles included in the study. In the data analysis stage, the content was categorized according to the similarities of the barriers and challenges identified. Finally, in the interpretation of the results, a reflective and critical analysis of the content was conducted, summarizing the content into themes for analysis [17].

## Results

**Fig. 1:**
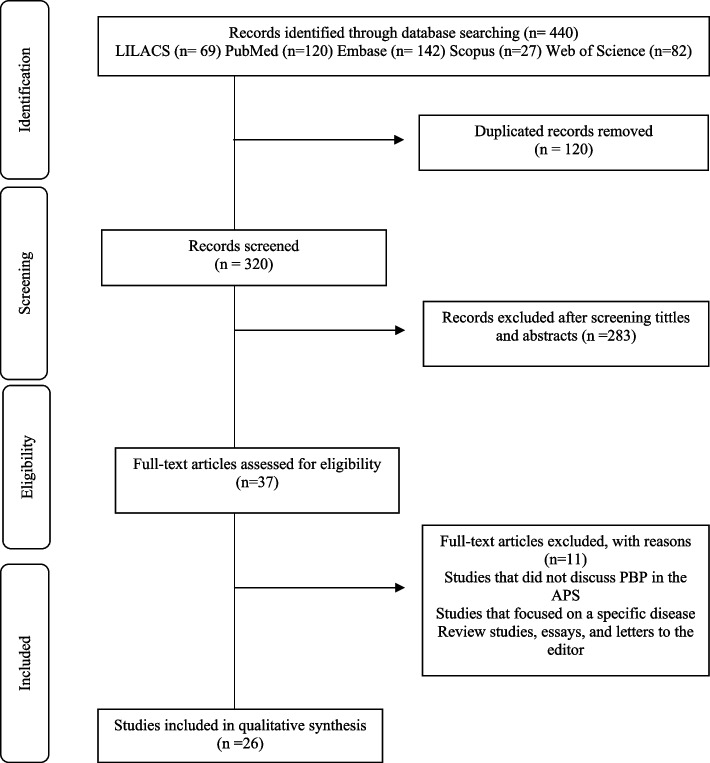
Flowchart of study selection

A total of 440 publications were identified in the databases. After excluding duplicate studies (*n*=120) and those that did not answer the guiding question (*n*=283), 37 studies were read in their entirety. Out of these, 11 were excluded as they did not meet the eligibility criteria. The final sample consisted of 26 studies (Fig. 1), with the majority being published in the past two decades and conducted in high-income countries (HICs), primarily in the United States of America (*n*=13). Furthermore, a significant proportion of these studies were case studies focused on the medical profession (Table 2).

**Table 2:** Description of the primary studies included in the integrative literature review according to the lead author, year, country, objective, population, and type of study

**Authors**	**Year**	**Country**	**Objective**	**Population**	**Type of Study**
Advocat et al. [18]	2015	Australia	Describe a new three-way partnership between a health authority, a primary care organization and a university in the suburbs of southeastern Melbourne. The partnership, known as SAPCRU, is a potential model for organizations that seek to bridge the gap between research and the real world.	Representatives of the partnership organizations (*n*=9)	Case study
Anderko et al. [19]	2005	USA	Describe the experiences of the Community Nursing Homes with PBRNs in primary health care research and highlight the need for research on community-based primary care to approach the health disparities experienced by large populations in the United States.	Community nursing homes (*n*=8)	Case study
Bodenheimer et al. [11]	2005	USA	Alert researchers to pitfalls they may face when working with the double motive of research and improving the practice.	Practice-based Research Networks (*n*=17)	Case study
Cole et al. [20]	2014	USA	Describe the main non-technical challenges that the academic implementation team encountered during the project. The objective is to describe approaches that can be used to effectively tackle these challenges.	Primary care clinics (*n*=9)	Implementation study
Davies et al. [21]	2002	UK	Evaluate the interest level for research among nurses who work in Essex and East London, United Kingdom; (2) identify the research priorities for nurses in the practice; and (3) explore the factors that facilitate or hinder the development of practice-based nursing research.	Nurses (*n*=1054)	Mixed methods
Delaney et al. [22]	2012	USA	Develop electronic health records software to facilitate clinical primary care studies and explore barriers to the adoption of the prototype by PBRNs in the United States.	NR	Case study
Dolor et al. [2]	2011	USA	Develop an open-access site providing adaptable resources to facilitate best practices in research.	Researchers (*n*=55)	Observational description
Farland, et al. [23]	2012	USA	Describe the steps taken by UT Pharm Net using a structure of principle strategies and directives to successfully develop a PBRN in various areas of interdisciplinary primary care practice.	Pharmacy students and residents (*n*=NR)	Observational description
Heal et al. [24]	2008	Australia	Describe the process of conducting a successful randomized controlled trial in a PHC environment and identify facilitating factors and barriers to investigating the effect of letting sutures be damp and not covered in the first 48 hours after small excisions.	PHC users (*n*=857)	Description of a randomized controlled trial
Hoffmann et al. [25]	2015	USA	Describe a qualitative evaluation of the experiences of primary care physicians and the clinical team that participated in various Practice-based Research projects.	Doctors, advanced practice nurses, nurses, clinic managers/directors, physician’s assistants, lab techs, receptionists and admin staff (*n*=53)	Qualitative study
Holden, et al. [26]	2012	Australia	Evaluate the impact of a research training approach for primary care teams using a validated quantitative measurement for research training for the individuals, team and organization.	Multidisciplinary teams (*n*=8)	Non-randomized study
Hudson et al. [27]	2006	USA	Provide a model to recruit community-based primary care clinics with minority physicians for research studies.	Primary care clinics (*n*=18)	Intervention study
Loskutova et al. [28]	2018	USA	Present a detailed case study of the recruitment methods and results used in a large practice-based study.	Primary Care Clinics (*n*=25)	Case study
Macfarlane et al. [29]	2005	UK	Identify the main developmental and environmental structural characteristics associated with successful and sustained involvement in research and inform a national strategy for primary care research training.	Lead clinical doctors (*n*=7), clinical doctors (*n*=4), nurses (*n*=10), research coordinator (*n*=1) and managers (*n*=6)	Qualitative study
Mash [30]	2020	South Africa	Describe the experience of implementing the Stellenbosch University Family Physician Research Network (SUFPREN)	Family physicians (*n*=25)	Experience report
Michalec et al. [31]	2013	USA	Understand the perceived restrictions on primary care practices from being involved in studies with perspectives on the micro, meso and macro levels.	Professionals from 5 Primary Care Clinics (*n*=17)	Qualitative study
Mold et al. [32]	2012	USA	Discuss the potentials of a coordinating center for multiregional PBRN studies based on 2 recent studies.	Coordinating centers for research based on primary care practice (*n*=NR)	Observational description
Morténius [33]	2014	Sweden	Describe, accompany and evaluate a primary care campaign based on strategic communication designed to increase health professionals’ interest in R&D over time.	Members of the PHC team (nurse, medical secretary, admin staff, midwife, physiotherapist, occupational therapist, dentist, psychologist, physician) (*n*=846)	Cohort study
Nagykaldi et al. [34]	2008	USA	Describe how the technology Access Grid (AG) was used by a PBRN.	American PHC practice-based research networks (*n*=NR)	Observational description
Planas et al. [35]	2019	USA	Describe the perceptions of a group of physicians who are part of PBR about: the development of a pharmaceutical that works with PBRN, aspects of the practice that can benefit from the collaboration with pharmaceuticals that are part of a PBR and benefits and challenges from the participation of the PBR members.	Physicians (*n*=15)	Qualitative study
Ponka et al. [8]	2020	Guiana, Sub-Saharan Africa, Malaysia, Nigeria	Explore the current risks or barriers to research training in PHC, identify the ongoing tensions that need to be resolved and offer solutions.	Low- and middle-income countries (*n*=5)	Multiple case report
Robitaille et al. [36]	2014	Canada	Describe an original and systemic recruitment process that was created to overcome the main barriers to enrolling family physician-patient pairs in Practice-based Research Networks.	Family physicians (*n*=276) and patients (*n*=276)	Observational description
Romani et al. [37]	2016	Bahrain, Egypt, Iraq, Jordan, Lebanon, Oman, Saudi Arabia, Syria and UAE	Explore the current status of academic research on primary care in Arab countries and investigate the barriers to its adequate implementation.	FCM academics in Arab countries (*n*=139)	Observational description
Soós et al. [10]	2010	Australia	Discuss key factors for establishing and developing the organizational structure of the Victorian Primary Care Practice-Based Research Network (VicReN) and describe the outcome measures used to evaluate the network.	Primary care professionals and academics (*n*=117)	Case study
Thandi et al. [9]	2021	Canada	Report recent descriptive discoveries about weaknesses, describe strategies for working in practice-based research and learning networks (PBRLNs) in primary care and share lessons learned to engage PBRLNs.	Physicians (*n*=109)	Participation-based descriptive study
Wasserman et al. [38]	1998	USA and Puerto Rico	Describe the establishment of a national network for pediatric primary care research to improve child health care—Pediatric Research in Office Settings (PROS)—and evaluate the progress of the network in reaching its goals.	Pediatric doctors and nurses (*n*=1400)	Case study

*NR* Not Reported

During the data analysis, six overarching themes and 15 subthemes related to the challenges of carrying out PC-PBR emerged. Among these challenges, difficulties regarding *research planning* were noteworthy, with issues ranging from excessive bureaucracy to challenges in planning and developing a research project. The *Engagement of health professionals in research* was recognized as one theme encompassing four different subthemes: lack of training and experience in scientific writing; difficulties with foreign languages; previous negative research experiences; and fears of negative impacts on the healthcare team, patients and productivity. *Challenges regarding knowledge translation* detail the difficulties in applying the knowledge acquired from one article to a change in daily work. *Infrastructure issues* are related to the location of the health services and how dispersed they can be in one area, the lack of technological tools and the little access to funding resources to sponsor more robust and long-term projects. Finally, a *weak relationship between universities and health services* can lead to little – or even no – collaboration between research institutes and PHC practices. The *lack of international partnerships* is finally presented as one main challenge for low- and middle-income countries (LMICs) since such collaborations could be helpful in building capacity for young research centers to address pressing issues in contexts where PHC is still very incipient (Table 3).

**Table 3:** Summary of findings on challenges for conducting PC-PBR

**Main Topic**	**Subtopics**	**Keys**
Research planning	Bureaucratic aspects/flows	Submission to and approval by the ethics committee [9, 11, 24, 35]
	Project preparation and development	Choosing the research question [8]
Engagement of health professionals in research	Research abilities	Lack of training [36, 37]
		Lack of experience with scientific writing [8]
		Ability and confidence to start and conduct studies [30]
		Difficulty with the language of the articles [33]
	Fears of professionals and management	Frustrating research experiences [27]
		Fear that the study will hinder the team and relationship with patients [36]
		Fear that the study will have a negative impact on patients [36]
	Organizational aspects	Lack of time to dedicate to research [8, 26, 30, 31, 35, 36]
		Heavy caseload [24, 37]
		Research activities overloading clinical tasks [37]
		Competing demands (care and scientific) negatively impacting productivity [28, 31]
		Institutional consent to the professional’s participation in a research project [28, 35]
	Incentives and advocacy	Little incentive for PHC research [8]
		Lack of interest, engagement and motivation for health professionals [9, 37]
		Lack of support for research from health services [37]
Knowledge translation	Application of knowledge	Difficulty in translating knowledge into health policies and practices [8]
		Lack of randomized studies estimating outcome measures of campaigns [38]
Infrastructure	Location and structure	Geographic isolation in remote and rural areas [35]
		Precarious physical structure to host a research group [9, 37]
	Technological resources	Irregular internet access [24]
		Differences in data-sharing systems [9]
		Unavailability of electronic records [37]
		Precarious access to software and statistical tools [8]
		Lack of adequate technology for sharing data [20]
	Funding	Limited financial resources to invest in infrastructure [20]
		Costs progressively increase as a research network grows [38]
		Shortage of financial resources to conduct studies, especially in low- and middle-income countries [37]
Relationship between universities and health services	Training	Offering of research courses and training is restricted to master’s and doctorate program norms [21]
		Shortage of qualified supervisors [8]
		Lack of interprofessional collaboration and education with a multidisciplinary approach [8]
	Integration of research and practice	Distance between health professionals and researchers [8]
		Universities and research centers maintaining a conservative view of the way to conduct studies [8, 26]
		Precarious link between universities and health services [26]
		Academic priorities do not reflect community need [8, 26]
		Lack of a common agenda between universities and PHC services [18]
Partnerships between countries	Exodus of researchers	“Brain drain” on different levels [8]
	International collaboration	Little international collaboration to conduct studies in developing countries [8]
		Lack of training to do research in developing countries [8]

The strategies listed in the articles included in this review were organized according to the challenges described in the previous section. The following were highlighted: suggestions related to creating a research agenda adapted to each reality; training strategies to develop research skills; sharing the results with all stakeholders involved, from participants to health managers and decision-makers; and the importance of creating networks for practice-based research (Table 4).

**Table 4:** Strategies for conducting PC-PBR

**Challenges**	**Strategies**
Research planning	Understand how your regional ethics committee works [19]
	Include all stakeholders in the study (professionals, researchers, patients, employees), from initial development to conducting the study [25]
	Consider the entire served population as a potential study population [27]
	Think proactively and create an agenda for studies based on your reality [29, 31]
	Identify national and international funding opportunities [8]
Engagement of health professionals in research	Hold trainings to develop research skills and share experiences [8]
	Initiate scientific activities with “small projects” [25]
	Involve patients in designing practice-based research projects [23]
	Guarantee allotted time in the professional schedule to develop studies [29]
	Advocate for studies to be done in PHC practice settings [8]
	Promote opportunities for collaboration among individuals [26]
	Encourage professionals to learn more about studies and reflect on their own practice [10]
	Involve different parties, especially governments, academic institutions, societies and funding institutions to promote the coordination of research efforts [37]
Knowledge translation	Plan the stages involved in knowledge dissemination [30]
	Guarantee dialog with health policymakers and identify priorities and particularities of implementation in countries’ different development contexts [8]
	Seek out the best ways to implement the results of studies [38]
	Share the results with study participants, professors, academics, health professionals and municipal managers [25]
	Identify opportunities to speed up the translation of discoveries into practice [2]
Infrastructure	Connect universities and research institutes to local practice-based research networks [8]
	Work collaboratively with all parts of the network and establish clear priorities [19]
	Use secure technology to identify potential patients and facilitate communication between information systems [23]
	Develop and use coordinating centers as a way to strengthen the PBRN research infrastructure and increase the reliability and generalization of the study results [8]
Relationship between the universities and practice	Involve the community and understand local needs [8]
	Bring research networks closer to PHC professionals [22]
	Strengthen the interaction between universities, research institutes and practice to guarantee joint ownership of the research [8]
	Establish international and multidisciplinary collaborations [38]
	Consider the possible conflicts between research and the change in practice [11]
	PBRN projects should be led by professionals or professors who carry out healthcare activities in PHC services [23]
	Establish practice-based research networks, contributing to the increase of relevant research on the local level and building up research capabilities [30]
	Define the roles of members from academia and health services and select a coordinator who is responsible for the research project [30]
Partnership between countries	Explore different contexts of practice to enrich your research, establishing comparisons [8]
	Defend the ability to research in all countries, including low- and middle-income ones [8]
	Create contact networks between researchers from different countries [8]
	Explore already existent collaboration opportunities [8]

### Challenges and strategies for conducting PC-PBR

#### Research planning

In this domain, a series of challenges related to designing a research plan are combined, such as developing and refining a research question, designing a strategy for data collection and data analysis, writing and submitting a proposal to the ethics board committee and the amount of time it takes to obtain the approval to start the project [8, 9, 11, 18, 30, 32, 35]. The time needed to carry out and conclude a study is often very different from the amount of time needed to make decisions in health care. Conducting a study with the length of time necessary to meet the needs for the transformation of health services is a difficult task, since managers and decision-makers may have more immediate expectations and hope for quick solutions to their problems [8]. To overcome this limitation, it is important that all stakeholders (managers, patients, health professionals, and researchers) are involved in the study, mainly to facilitate the understanding of the steps that one study needs to go through until its publication [9, 18, 38].

#### Engagement of health professionals in research

Some decision-makers and health managers fear that a research project can cause trouble in the way that a health facility is used to operate, impairing its productivity or even hindering the patients’ trust in the health service [8, 18, 21, 30, 31, 35, 36]. In addition, many managers see research projects as less important than practice, without acknowledging the possible benefits of research on patient care [28]. Researchers must bring these issues into debate with health managers and decision-makers so that barriers such as a lack of time dedicated to research, high caseloads limiting the time dedicated to research, and the need for institutional approval to allow professionals to participate in research projects can be overcome [26]. If this is not done, it will be difficult to create a routine of knowledge production and innovative research that integrates healthcare professionals, patients and researchers to create robust scientific evidence with an impact on the workplace, patient care and the quality of the services provided.

#### Knowledge translation

This theme, which is known as integrated knowledge translation in the current literature [39], involves the processes of generating, sharing, and applying knowledge, not necessarily in that specific order [8, 32]. In theory, carrying out PC-PBR is a powerful resource to make knowledge translation happen, since research questions are created to answer local needs, relying on the participation of professionals – and sometimes the patients – in practice [32].

However, one of the barriers to knowledge translation lies in the difficulty of adapting the knowledge to contexts that are distinct from those where one study was held, e.g., results from HIC being translated to LMICs. This reinforces the need to involve all stakeholders in the stages of designing the project to describe the aspects of the context where the research will be held, outlining this information in the discussion section of the article as well, making it easier for the reader to understand its external validity [2, 8, 30, 38].

The long time span for the publication of the study results in scientific journals, in addition to the high rejection rate, are factors that further delay the process of knowledge translation. Considering the dynamic nature of primary care services, studies should have a broad plan to disseminate results, to implement the evidence in a timely manner [30].

#### Infrastructure

Challenges related to infrastructure are frequently found in PC-PBR studies, from the distance between primary care services in rural settings and the difficulty of reaching some services to the often lack of technology resources, such as internet access, and patients’ electronic records [8, 9, 20, 23, 32, 35].

The lack of reliable, sustainable, and systematic funding for PC-PBR research activities is the main obstacle to overcoming these infrastructure limitations and promoting the creation of PC-PBR [8, 10, 19, 23, 27, 31, 35]. Like every research initiative, PC-PBR needs to be supported with adequate and constant funding. For that reason, researchers must remain attentive and updated to identify funding opportunities [18].

Healthcare services produce a large volume of data every day. Information about healthcare procedures, prescriptions, patient profile, and all sorts of interactions between the patient and their healthcare providers. However, the quality of the information input and the way it is stored can limit its use [9]. It is essential for managers and stakeholders to verify how these data have been used, not only how practitioners use them for patient management but also for research, surveillance, and accountability [19, 23].

Confidential information should be strictly and safely handled so that no patient information becomes public, allowing its use for research with no harm to the patient or for the practice [34]. For this purpose, all parties using these data must agree to a common commitment across the PC-PBR network to develop and implement research programs. Ideally, the research priorities should be established by the researchers and managers, with a clear evaluation of the capabilities of each practice, the information systems available and the whole network. When used appropriately, these real-world data can generate new knowledge from practice to improve patient care [18].

#### Relationship between universities and health services

Some studies highlighted the strains of integrating universities and health services [8, 18, 21]. The distance between these two scenarios can be explained by several factors: (a) the fact that academic priorities may not reflect the needs of the communities [8]; (b) weak connections between academia and primary care services [19]; (c) the lack of a mutual agenda between them combining common interests [25]; (d) the distance between researchers and health professionals [8]; and (e) the restricted access to specific research training courses run by universities, apart from formal master’s and doctorate courses [21]. Such training courses are usually offered during workdays, which limits the participation of those who work full-time as health care providers. Offering postgraduate courses in research aimed at health professionals that take advantage of the students’ experience to generate relevant research questions and new knowledge for healthcare could be transformative both for universities and health services. However, gathering individuals who traditionally work in different sectors is not easy. In addition, creating organizational structures that support primary care-based studies can demand financial resources, time, and people, which are not easily available [29].

Among the strategies found in the articles to overcome this challenge, it is important that the research questions arise from practice and that the roles of researchers, academics and health professionals are well-defined within the group. In addition, it is important to select a coordinator responsible for managing the research project and the tasks that need to be executed [30, 34].

Implementing PC-PBR can bring results both for practice and academia, bringing together different professionals to achieve a common goal of improving patient care. Strengthening the interaction between academia and primary care services can help to promote the sustainable development of research projects in which health professionals can develop innovations in health care that can be studied and tested, creating a virtuous cycle beginning with raising questions from practice, conducting experiments, finding results and producing evidence that can serve the purpose of improving patient care and the health of the population [19].

#### Partnerships between countries

Despite this being a topic addressed in only two of the articles under analysis, promoting international partnerships can be a solution to many of the challenges mentioned here. However, such collaborations are not yet a reality for many countries. There is a shortage of international initiatives to promote research courses and training to bring together mentors from HIC and young researchers from LMICs and provide direction for conducting studies in contexts with few resources [8].

In addition, many professionals from LMICs who are involved in studies or education abroad end up migrating to other countries, contributing to the so-called “brain drain” of skilled professionals and worsening the inequality in scientific production between HICs and LMICs.

Addressing research projects within the local context and exploring opportunities for international collaboration is important enough to foster PBR and guide health professionals in places where universities and research institutes are not yet established. Moreover, it is important to consider the epidemiological profile, cultural aspects, and social determinants of health in every scenario involved when an international collaboration is planned. The different contexts of practice can enrich the research and establish comparisons that can be decisive for international scientific advancement [8].

## Discussion

The challenges and strategies for the implementation of PC-PBR indicate operational, structural, and political issues. One of the key aspects learned about planning a PC-PBR study is to identify and include all stakeholders (patients, employees, doctors and administration) in the development phase of the project, allowing for discussions about the study design and its implementation phases. This approach must become an integral part of the study, being comprehensive to addressing barriers to participation, obtain data, analyze and interpret the results and, finally, discuss its findings and implications. Additionally, planning data collection that demands little effort from health professionals can strengthen the study’s realization and the involvement of everyone.

In this context, it is important to emphasize that all challenges are even more pronounced in LMICs. In this regard, efforts are being made towards decolonization [40], encouraging research that validates the context and perspectives of local thinkers, thereby expanding the discussion to generate and incorporate evidence into real scenarios that value the knowledge of communities, healthcare professionals, policymakers, and researchers in LMICs. Therefore, the present study aimed to synthesize the challenges and strategies that underlie this discussion, but a gap was identified in terms of the production of this discussion in LMICs.

To address the issue of limited international collaborations in LMICs, it is crucial to explore targeted implications and strategies to surmount this constraint. Some viable strategies involve providing training and education in cultural sensitivity, thereby enhancing the efficacy of these partnerships. While international collaboration typically prioritizes partnerships with high-income countries, LMICs can also explore collaborations with other LMICs. Sharing knowledge, best practices and resources with neighboring countries facing similar challenges can result in mutually advantageous outcomes.

PC-PBR only happens if the professionals who are directly involved in patient care and health service management are integrated as part of the team of researchers, not just as the subjects of the research [8, 36]. Although it is a great challenge, training healthcare professionals to conduct research in primary care is fundamental for the success of these projects [23, 24].

Alternative research approaches, such as implementation research, have advanced and grown as new strategies to reduce the gap between research and practice, mainly because they systematically approach the factors that contribute to this gap, understanding the context and identifying barriers and solutions for delivering sustainable and effective health care [41]. Thus, to make progress in overcoming these structural barriers it is important to understand the essential pieces of the research process, without which a project will likely die prematurely. One of these elements is the minimal infrastructure needed for PC-PBR research projects to be long-lasting and sustainable [9, 23].

The studies under analysis point out that the most promising way for this to happen is through collaboration between primary care services, universities, and research institutes. In addition, these collaborations can provide training in research skills for health professionals, creating an environment conducive to exchanging experiences, ideas, and questions about the practice. All these suggestions will help to create a research agenda oriented toward solving real issues related to taking care of patients in primary care, which is the main objective of conducting PC-PBR [8].

The distance between universities and primary care settings is recurrently cited. This issue reinforces the idea that there is a place where knowledge is produced (universities and academia) that is different from the places where health care occurs. In other words, primary care is seen as a place where scientific evidence produced by academia is put into practice.

Conducting scientific research within primary care practices is innovative and can create ruptures and conflicts when it affects the way the job is done or when it takes people out of their comfort zones. By placing health professionals—and at times, patients—as agents of research production, PC-PBR can change the way new knowledge is produced. If knowledge is traditionally produced in academia and then taken as a truth by the place where patient care occurs, PC-PBR can not only generate new knowledge to change professional practice but also bring new evidence to change the way academia works, guiding new research that is better aligned with reality [34].

In some countries, a more horizontal construction of new evidence and knowledge translation can be seen between academia and healthcare practice. In Australia, for example, PBR protocols are designed to build a sustainable collaboration between a PBRN and an Advanced Center of Research and Translation in Health to build a research platform for planning, conducting and translating research evidence to improve care across the healthcare spectrum [42].

Aligned with the need for partnership between universities and practices, international collaborations are also an opportunity to guide professionals in places where universities and research institutes are not yet established. Cases such as Australia and New Zealand, where two PBR networks were established to encourage research in the area of osteopathy, show that PBRN has the potential to facilitate the access of professional researchers and clinics that are interested in collaborating with clinical tests and, thus, offer the scientific community an opportunity to conduct research with different methodologies in diverse contexts [42].

Regarding the difficulties in engaging health professionals in PC-PBR, some examples listed in the articles were little experience in scientific writing, difficulties reading articles in foreign languages, limited self-trust and lack of training to start and conduct studies. Thus, studies recommend that universities and research institutes organize training courses to develop research skills and exchange experiences to determine shared research priorities [8].

Although essential, the development of research skills is not enough for professionals to engage with and incorporate studies into their places of practice. For PC-PBR projects to advance, leadership is necessary to influence policymakers and managers and advocate for studies to be directly connected with the practice where health care happens.

The majority of the selected studies highlighted the medical category in the discussion about PBR. However, it is important to expand the professional composition of PC-PBR beyond and consider other categories to organize more participative and multidisciplinary studies. All health professionals must be invited to interact and collaborate with scientific activities and implement new projects. The inclusion of all health professionals, including community health workers, nursing assistants, and dental hygienists, who are commonly found in LMICs, can improve the development of research projects that will better take into consideration the patients’ and the territory’s needs [8].

Implementing PC-PBR goes beyond research production, since the results of the studies produced by researchers, health professionals, users and managers, in addition to the lessons learned, are shared with the health service where the study was held, bringing greater transparency to the entire process and motivating more health professionals to actively participate in future research projects [38].

### Limitations

This review was limited to the literature that reported lessons learned and experiences conducting PC-PBR since few empirical studies with primary data from practice were found. Additionally, there is little representation from LMICs. This limits the conclusions of this review to the contexts described herein, i.e., HIC, where PHC already has a solid structure and a robust research production. Exploring studies performed in PC-PBR networks and identifying their strengths and weaknesses would be a step forward in this sense, but it would demand greater operational efforts. However, this is the first review that is necessary for the advancement of primary care research mainly in LMIC.

## Conclusion

The challenges for implementing PBR are similar in the contexts analyzed, showing that turning one place that was originally designed for delivering primary care into a place of knowledge production is not a trivial task. The benefits depicted in the studies show that transforming the traditional methods of knowledge production and translation through PC-PBR can generate a virtuous cycle, providing criticism and reflection about the practice and generating innovations and new knowledge to improve healthcare and patients’ health and well-being.

Additionally, the found strategies point to the need for lasting and systemic actions involving health managers, decision-makers, academics, different types of health professionals and patients, aiming to transform PHC practice in the long term. Despite being more the exception than the rule, PC-PBR has the potential to transform a PHC system that is still under development into an innovative, socially accountable, more comprehensive, accessible, and patient-centered healthcare approach. Furthermore, recognizing the transformative potential of PC-PBR, it becomes imperative to explore strategies for scaling these practices and approaches, ultimately having a broader and more profound impact on the entire primary healthcare system.

## Data Availability

All data generated or analyzed during this study are included in this published article.
